# COVID-19 control during a humanitarian crisis; the need for emergency response at the Thai-Myanmar border as an alternative channel

**DOI:** 10.1186/s41182-021-00323-1

**Published:** 2021-05-07

**Authors:** Jun Kobayashi, Nanae Aritaka, Ikuma Nozaki, Aya Tabata, Shinichiro Noda

**Affiliations:** 1 Department of Global Health, Graduate School of Health Sciences, University of the Ryukyus, 207 Uehara, Nishihara, Nakagami-gun, Okinawa 903-0215 Japan; 2 Japan Association for Mae Tao Clinic, 3-21-7 Ginowan, Ginowan, Okinawa 901-2211 Japan; 3 Bureau of International Health Cooperation, National Center for Global Health and Medicine, 1-21-1 Toyama, Shinjuku, Tokyo, 162-8655 Japan

**Keywords:** Thai-Myanmar border, Refugee, COVID-19, Alternative channel

## Abstract

Following the coup in Myanmar, humanitarian assistance, including coronavirus disease 2019 (COVID-19) control, must be implemented on the Thai-Myanmar border in the framework of international cooperation. The actual number of refugees was expected to increase in the Karen state at the end of March 2021, and they are at risk of contracting COVID-19 as they live in overcrowded conditions without access to basic sanitation. The global community has been hesitant to provide direct support because of fearing that such support would benefit the military. To reach this most vulnerable population, further strengthening of support through the Thai-Myanmar border as an alternative channel that was used before Myanmar's democratic transition in 2011 is necessary.

## Risk of COVID-19 infection among refugees on the Thai-Myanmar border

Following the February 1st coup in Myanmar, the number of refugees likely preparing to cross the Thai-Myanmar border is expected to increase. Not only is the military unilaterally persecuting the general population, as is occurring in Yangon, but a state of war is likely beginning between the national army and ethnic minorities. From information received from the Mae Tao clinic, a health facility established by refugees from Myanmar to Thailand in 1989, more than 10,000 people have fled to the Karen and Karenni states from cities including Yangon and Mandalay, and most seem to be activists, non-governmental organization leaders, and participants in civil disobedience. Further, intensified attacks in ethnic areas since December 2020 have internally displaced over 8000 people according to the Free Burma Rangers [1]. After Myanmar’s military began airstrikes in the Karen state on 27 March (Armed Forces Day), the Karen Information Center reported that at least 10,000 local villagers fled into the jungle, and more than 3000 had crossed into Thailand by 29 March [2]. However, Thai authorities reportedly forced over 2000 refugees back to Myanmar [3] despite danger of further attacks. These internally displaced persons would definitely be expected to require immediate humanitarian aid.

Although World Health Organization (WHO) reported that these vulnerable people are generally at low risk of transmitting communicable diseases to host populations [4], they are at risk of contracting diseases, particularly COVID-19, due to overcrowded conditions lacking access to basic sanitation [5]. In humanitarian settings, access to health-care services is often compromised due to medicine shortages and lack of health-care facilities. Additionally, administrative, financial, legal, and language barriers hinder their access to the health system.

Should COVID-19 spread to the refugees’ border camps, it would be inhumane to leave them unattended, and ordinary activities of conventional medical facilities would likely come under great pressure. Further, discrimination and prejudice would deprive the refugees of work opportunities and exacerbate domestic violence and human trafficking [6].

## The need for emergency response through an alternative channel

We recommend the provision of refugee assistance within an internationally coordinated framework at the Thai-Myanmar border that includes COVID-19 response and care. In particular, Japan can play a certain role because of its experience in providing humanitarian assistance to Myanmar both before and after the democratic transition in 2011. Although there are currently no official reports of increased cross-border refugees on the Thai side, many ordinary residents on the Myanmar side are already hiding in the jungle to avoid persecution by the Myanmar military, and the risk of these people becoming cross-border refugees is high.

Global society is willing to provide direct humanitarian support to Myanmar, but the Myanmar people are refusing all international support that might benefit the military, including any kind of official developmental assistance. Thus, we believe that further strengthening of support through the Thai-Myanmar border as an alternative channel, which was used when Myanmar was under sanctions, is necessary to reach the people in this most vulnerable and difficult situation. To aid Indochinese refugees, we have been working with Thai and international societies [7] even though the Thai government does not officially recognize refugees. This long-time cooperation with other international donors through civil society includes the Japan Association for Mae Tao Clinic. We believe that the global society including Japan can play a pivotal role in this critical situation.

Although the risk of COVID-19 spreading across the border is unclear, it is probably not low. In January 2021, just before the military coup, approximately 300 to 700 new cases were confirmed daily in Myanmar, and the number of cases totaled 140,145 [8]. However, due to the coup itself and civil disobedience of medical professionals, little COVID-19 testing has been performed after February and especially in March; thus, obtaining accurate information would be difficult. At the end of March in Tak Province, which borders Myanmar, 126 of the 344 confirmed cumulative case cases of infection were acquired from abroad [9].

Even though Thailand has achieved some success in COVID-19 control, this issue must be addressed within the framework of international cooperation beyond the health sector. Since before Myanmar’s democratization in 2011, Japan has continued to provide support to the health and education sectors and has worked with the Ministries and Myanmar government to provide humanitarian assistance. Furthermore, Japan’s medical assistance to Indochinese refugees in the 1980s was pioneering in health sector support in Japan. We expect Japan to continue to work with Thai and international civil societies to address this potential current crisis at the Thai-Myanmar border.

## Data Availability

Not applicable. The manuscript does not contain any data.

## Abbreviations

COVID-19: Coronavirus disease 2019
WHO: World Health Organization
